# Assessment of energy and nutrient intakes among undergraduate students attending a University in the North of England

**DOI:** 10.1177/02601060221096932

**Published:** 2022-06-20

**Authors:** Helen R. Martin, Deborah A. Pufal, John Stephenson

**Affiliations:** 1 Department of Service Sector Management, 7314 Sheffield Hallam University, Howard Street, Sheffield, S1 1WB¸ United Kingdom of Great Britain and Northern Ireland; 2 School of Applied Sciences, 218224 University of Huddersfield, Queensgate, Huddersfield, HD1 3DH, United Kingdom of Great Britain and Northern Ireland; 3 School of Human & Health Sciences, 14270 University of Huddersfield, Queensgate, Huddersfield, HD1 3DH, United Kingdom of Great Britain and Northern Ireland

**Keywords:** Undergraduate students, NDNS, energy and nutrient intakes, DRVs

## Abstract

**Background:** Unhealthy diets are typical of university students and are often thought to be unrepresentative of the general population. The main aim was to determine the energy and nutrient intakes of a large cohort of undergraduate university students; and to compare to gender-specific dietary reference values (DRVs) and nutrient data from the general population. **Methodology:** Data was collected from 639 university students aged 18–24 years who completed 4-day diet diaries. The energy and nutrient intake was determined and percentage energy values calculated and compared with dietary reference values (DRVs) and the National Diet and Nutrition Survey (NDNS) and Family Food Statistics. Logistic regression methods were used to identify micronutrients functioning as predictors of exceeding DRVs. **Results:** Energy intakes were lower than the DRV. The percentage total energy values for protein, fat, saturated fat and carbohydrate exceeded DRVs but the percentage energy from alcohol was below the maximum 5%. The DRVs were met for vitamin C, thiamin, and sodium/salt. Iron and calcium intakes were met in males but not in females. Intakes for fibre and vitamin A were below the DRV. Student data was comparable to the NDNS, with the exception of alcohol, fibre, vitamin A, calcium and sodium/salt, which were all lower than the NDNS. **Conclusions:** This study contradicts the stereotypical assumption that students are following a high energy, fat, saturated fat, total sugars, salt and alcohol diet compared with the general population.

## Introduction

University often represents a transient time for students. Many have moved away from home for the first time, resulting in increased responsibility and independence regarding a healthy lifestyle and food choice (Colić Barić et al., 2003; Li et al., 2012; Nelson et al., 2008). It is widely assumed that university students consume large amounts of alcohol (Davoren et al., 2016) and subsist on diets based on convenience (fast food, ready meals); with excessive levels of fat, particularly saturated fat, salt and sugar; and low levels of fruit/vegetables, fibre and complex carbohydrates, indicative of a high calorie/energy diet (Hilger et al., 2017; Nelson et al., 2008). Evidence exists that many students, particularly those who had moved away from home rather than still living in the family home, gain weight and typically adopt less desirable eating habits, such as the consumption of more ready meals and takeaway meals and fewer home-cooked meals, when first enrolling at university (Hilger et al., 2017; Li et al., 2012; Papadaki et al., 2007). This may be linked to poor eating habits, lack of exercise and the stress of adapting to a new environment, which can often trigger over-eating.

### Barriers to healthy eating

Barriers to healthy eating in the student community have been identified as a lack of time due to studies, lack of healthy food at university canteens, and the high cost of healthy food (Hilger et al., 2017; Musaiger et al., 2014; Pelletier and Laska, 2012) in contrast to the cost of fast foods and ready meals, which are frequently sold in special price deals. This is compounded by a lack of budgeting skills and limited financial resources (Nelson et al., 2008), increased availability of convenience and fast foods (Boone-Heinonen et al., 2011) and limited cooking ability (Nelson et al., 2008; Papadaki et al., 2007). However, intervention studies have found that providing food on campus that was cheaper, more varied and at a higher quality resulted in healthier food habits (Davis et al., 2009; Guagliardo et al., 2011; Michels et al., 2008).

Additional barriers to healthy eating are the erratic eating habits practised by some students who do not adhere to strict meal patterns; for example, eating late at night. It was found that the number of meals that students ate was negatively correlated with body mass index (BMI) (Breitenbach et al., 2016), indicating that students who ate more meals had lower, optimal BMI values. This suggests that the consumption of regular meals is associated with lower rates of snacking, which tend to be high in fat, saturated fat, sugar and salt. A previous study reported that about one quarter of students miss breakfast on one or more weekdays (Hilger et al., 2017). Lack of breakfast is likely to increase consumption of snacks. Other reasons for an individual's particular food choice (for students, and other groups) are: life experiences (social settings, cultural criteria); psychological and physiological traits; personal preferences; beliefs; expectations regarding food choice (Furst et al., 1996; Pei-Lin, 2004).

## Aims

This study aimed to determine the energy and nutrient intakes of undergraduate university students; and to compare these values to gender specific dietary reference values (DRVs) and nutrient data from the general population.

## Methods

### Data collection

Data was collected between 2010 and 2017 from 4-day diet diaries (including three weekdays and one weekend day) completed by second year undergraduate pharmacy students from a university in the north of England, UK. Participating students recorded all food and drink consumed throughout the day in the diaries. Detailed information was required for specific food types; for example: whole, semi-skimmed or skimmed milk. Portion sizes were estimated using the Food Atlas (Nelson et al., 1997).

### Statistical analysis

Dietary data was analysed, collating data over the entire period of analysis. Daily mean intake of total energy, carbohydrates (including total sugars), fat (including saturated fat), protein, fibre, vitamin A, thiamin, vitamin C, iron, calcium, sodium and alcohol was determined. Nutrient content of diets was evaluated using nutrimen, a professional web-based dietary analysis software, using the McCance & Widdowson composition of foods integrated data set of 2015 (Roe et al., 2015), based on UK food composition tables (www.nutrimen.co.uk). Percentage total energy values for carbohydrates (including total sugars), fat (including saturated fat), protein and alcohol were calculated.

Nutrient data was also compared to NDNS data. The NDNS rolling programme is a continuous cross-sectional survey providing information on food consumption, nutrient intake and nutritional status of the general population in private households in the UK. Where data from the NDNS was not available, data from the Family Food Statistics 2016/17 were used (DEFRA, 2018). Nutrient data were also compared to the UK DRVs by age and gender (Dept_of_Health, 1991).

Total energy intakes were compared to the estimated average requirement (EAR) (SACN, 2011). Carbohydrates (including total sugars), fat (including saturated fat), protein and alcohol were compared to reference percentage total energy values. Protein and micronutrients were compared to the adult reference nutrient intake (RNI); a value two notional standard deviations above the EAR. The lower reference nutrient intake (LRNI) is the value two notional standard deviations below the EAR.

Participants were categorised as exceeding or not exceeding gender-specific DRV and NDNS data for: total sugars; vitamin A; vitamin C; iron; sodium. Logistic regression analyses were conducted to assess the effect of nutrient intake on the probability of exceeding amounts using known relationships between nutrients, calcium, fibre, vitamin C, saturated fat and protein.

### Ethical approval

Permission to conduct the study was provided by the School Research Integrity and Ethics Committee in the university in which the study was based (SAS-SREIC 12.7.19-2). Confidentiality was maintained as no personal data was collected.

## Results

A total of 639 undergraduate students completed the diet diaries within the study period. The sample comprised 385 females (60.3%) and 254 males (39.7%); covering an age range of 19 to 24 years. 74% of the sample originated from BAME (black, Asian and minority ethnic) groups.

### Total energy intake

The mean total energy intakes were 6.0 MJ (SD 2.1 MJ; range 1.5-18.9 MJ)/1436 kcal (SD 497.5 kcal; range 359–4524 kcal) in females and 8.5 MJ (SD 3.2 MJ; range 2.3 MJ to 26.9 MJ)/2025 kcal (SD 769.6 kcal; range 547 to 6430 kcal) in males. The total percentage energy values in both genders, with corresponding NDNS and DRV values, are summarised in Table 1. In both males and females, the primary source of energy was from carbohydrate (around 50% for both genders) and total fat (around 35% for both genders). These values are comparable with the DRVs.

**Table 1.** Comparison of total percentage energy in female and male students with NDNS data and DRVs.

Source	Female intake (%)		Male intake (%)		
	Female (*n* = 385): mean (SD)	NDNS	Male (*n* = 254): mean (SD)	NDNS	DRV
Protein	16.6 (4.32)	16.7	17.8 (5.43)	17.0	15.0
Fat	34.8 (6.65)	33.8	34.6 (6.94)	32.6	33.0
Saturated fat	11.4 (3.48)	12.2	11.1 (3.28)	11.6	10.0
Carbohydrate	50.4 (7.02)	46.2	48.5 (8.24)	45.1	47.0
Alcohol	1.23 (3.40)	3.2	1.94 (4.65)	5.2	5.0
Total sugars	18.5 (6.50)	nd^*^	18.2 (7.98)	nd^*^	18.0^**^

* nd NDNS data not available for total sugars.

^**^ Based on the reference intake for food labelling (FSA, 2013).

### Nutrient intake

Protein, fibre and micronutrient intake, with comparative DRVs and NDNS data, is summarised in Table 2. Mean protein intake exceeded RNI values in both genders; however, female protein intake was less than NDNS values. Mean dietary fibre intake was lower in both genders than NDNS values and the SACN recommendations (SACN, 2015).

**Table 2.** Nutrient intake in female and male students with NDNS data and DRVs.

Nutrient	Female				Male			
	Intake	RNI	%RNI	NDNS/FF^*^	Intake	RNI	%RNI	NDNS/FF^*^
Protein g/d	58.3 (22.2)	45.5	130	66.6	89.9 (53.1)	55.0	211	87.4
Fibre g/d	12.0 (4.9)	30^$^	40.3	17.4	12.7 (6.3)	30^$^	42.3	20.7
Vitamin C mg/d	70.1 (67.8)	40	175	81^*^ (203%)	84.2 (107.1)	40	211	81^*^ (203%)
Vitamin A μg/d	454.9 (427.3)	600	76	825(138%)	578.5 (521.7)	700	83	921 (132%)
Thiamin mg/d	1.1 (0.6)	0.8	138	1.7^*^ (213%)	1.5 (0.8)	1.0	150	1.7^*^ (170%)
Iron mg/d	8.4 (3.4)	14.8	57	9.3 (63%)	13.4 (20.6)	8.7	154	11.6 (133%)
Calcium mg/d	598.5 (322.5)	700	86	746 (107%)	782.4 (414.3)	700	112	897 (128%)
Sodium mg/d	1937.5 (979.6)	1600	121	2720 ^**^ (170%)	2644.6 (1127.5)	1600	165	3640 ^**^ (228%)
Salt g/d	4.8	4	120	6.8^**^ (170%)	6.6	4	165	9.1^**^ (228%)

* Family Food Survey (DEFRA, 2018).

^**^ NDNS: Assessment of Dietary Sodium in Adults in England, 2014 (PHE, 2016).

^$^ SACN Carbohydrates and Health Report (SACN, 2015).

Vitamin A intakes were lower than the RNI in the sample; however, by contrast, vitamin C intakes were higher than the RNI. Thiamin and sodium intakes were also above the RNI. Differential effects across genders were observed for iron and calcium intake; with females consuming less than, and males more than, the RNI values for their gender.

### Analysis of relationship between nutrient intake and exceeding DRVs

Strong evidence for associations between nutrient intake and the probability of individuals exceeding DRVs were recorded in almost all cases; however, calcium intake was not a significant predictor of the probability of an individual exceeding DRVs for vitamin C intake. As expected, most associations were positive, with fibre and saturated fat intake revealed to have the most substantive effects on outcomes. The parameters of the logistic regression procedures conducted on all outcomes are summarised in Table 3.

**Table 3.** Summary of logistic regression parameters.

Outcome	Predictor(s)	*p*-value	Odds ratio (OR)	95% CI for OR
Probability of individual exceeding DRVs for Vitamin A	Fibre	<0.001	1.11	(1.07, 1.16)
Probability of individual exceeding guidelines^*^ for total sugars	Saturated fats	<0.001	0.958	(0.939, 0.977)
	Vitamin C	<0.001	1.010	(1.006, 1.013)
	Calcium	0.008	1.001	(1.000, 1.002)
Probability of individual exceeding DRVs for Vitamin C	Protein	0.020	1.012	(1.002, 1.022)
	Fibre	<0.001	1.188	(1.117, 1.263)
	Calcium	0.076	1.001	(1.000, 1.002)
Probability of individual exceeding DRVs for iron	Protein	<0.001	1.069	(1.057, 1.081)
Probability of individual exceeding DRVs for sodium	Protein	<0.001	1.047	(1.032, 1.062)
	Saturated fat	<0.001	1.124	(1.085, 1.166)

* Based on reference intake for food labelling (FSA, 2013).

## Discussion

Energy intake of both females and males in our sample was below EAR values (9.1 and 11.6 MJ /2175 and 2772 kcal respectively) and comparable with the NDNS (6.9 and 8.8 MJ /1632 and 2091 respectively). Females reached 66% of the EAR for total energy, whilst males reached 73%. This gender disparity is consistent with findings of previous studies, where the difference in energy intakes were linked to males consuming more fast food, fried potatoes/chips, pasta, rice, hard/soft cheese, red meat and sausages while females were consuming more salad/raw vegetables and fresh fruits (Hilger et al., 2017; Lupi et al., 2015; Mikolajczyk et al., 2009). Gender differences could be due to increased health awareness (Stock et al., 2001; Wardle and Steptoe, 1991; Yahia et al., 2015), better nutrition knowledge (Kresić et al., 2009) and more concern about body weight (Salameh et al., 2014; Wardle et al., 2006; Yahia et al., 2015) among females.

The reported low energy intakes might indicate that students in this study were likely to lose weight during their time at university. This contradicts findings of other studies; for example, reported weight increases of between 0.9 kg and 3.1 kg in the first year of university (Crombie et al., 2009; Deliens et al., 2013). Our study sample finding that 15.3% of females and 2.4% of males were consuming less than 1000 kcal/day is consistent with findings that weight loss is more prevalent in female students (Pérusse-Lachance et al., 2010; Salameh et al., 2014; Wardle et al., 2006). However, low energy intake in both genders could be due to under-reporting (Pryer et al., 1997), underestimation of portion sizes (PHE, 2018a; Rennie et al., 2007) and intentionally changing eating habits while completing diet diaries (Burke, 2015). Conversely, 0.8% females and 7.5% males were consuming over 3000 kcal/day. This could be due to higher levels of physical activity in males (NHS, 2018).

In our sample, percentage total energy values from fats and total sugars were higher than the DRVs (Dept_of_Health, 1991; FSA, 2013). This could be indicative of snacking, rather than eating less energy-dense larger main meals. Total energy from carbohydrates was slightly higher than the DRV (47%) for both genders (50.4% for females and 48.5% for males). The small differential effect across genders reflects a similar pattern seen in the NDNS.

The percentage of total energy from total sugars was in both females and males in our sample close to the 18% used as the reference intake on UK food labelling (FSA, 2013). Sources of total sugars include fruit/vegetables, milk and milk products, and free sugars. Our results indicated an association between exceeding the intake of total sugars with calcium, saturated fat and vitamin C. On this basis, milk and milk products may be providing males (for whom calcium RNI values were met) with a major source of total sugars, but not for females (for whom calcium RNI values were not met) who are known to be more likely to eliminate dairy products from their diet (Salameh et al., 2014; Yahia et al., 2015). In addition, the total percentage energy from fat and saturated fatty acids was slightly higher in both genders in our sample than the DRV, which could indicate a high fat intake. However, this was not supported by the total energy intake, which was lower than the EAR. Therefore, snacking on high fat, saturated fat and sugar snacks, could be the explanation rather than eating larger main meals including breakfast (Coulthard et al., 2017; Gaal et al., 2018; Laska et al., 2011). Furthermore, the high vitamin C status found in both genders could indicate a high intake of fruit and vegetables. However, this assumption was not supported with a high fibre intake; found to be lower than the recommendation of 30 g/day (SACN, 2015). This could indicate that a significant source of vitamin C was fruit juice (widely available around university campuses) rather than from fruit, which is high in free sugars and low in fibre. Despite fibre intake being low, there is a positive association with vitamin C (Table 3). Alternative sources of vitamin C are potato products, such as chips and crisps which, although are not rich in vitamin C and fibre, are frequently consumed by students (Hilger et al., 2017; Yahia et al., 2015).

The finding that fibre intakes in the study cohort were low compared with the SACN recommendation and NDNS values may be because breakfast, typically based on a source of fibre such as wheat-based cereals or wholemeal bread, is often missed (Teleman et al., 2015). Also, high fibre foods are not always the preferred option (Kuznesof et al., 2012; McMackin et al., 2013; Robinson and Chambers, 2018) and are perceived as more expensive (Kuznesof et al., 2012; McMackin et al., 2013; Robinson and Chambers, 2018).

Salt intakes in females were below the target of 6 g/d at 4.8 g, and males at 6.6 g (PHE, 2003). The current population average is 8 g/d (PHE, 2016). This could indicate that snacks high in fat and sugar, such as chocolate-based snacks and confectionary were consumed, rather than savoury snacks which would be likely to be high in salt. There is also an association between saturated fat and sodium, and between protein and sodium (Table 3). This indicates that consuming foods higher in saturated fat and protein are associated with a higher sodium content such as meat/poultry-based products.

The higher-than recommended intakes of protein may reflect the composition of meals in the UK; which are typically based on protein sources such as meat, poultry, eggs, cheese and pulses. However, other studies have found that meat and fish intake were low after matriculation (Hilger et al., 2017; Lupi et al., 2015) possibly for financial reasons (Guagliardo et al., 2011). The percentage total energy value for males was higher than that of females in the current study; possibly because of perceptions of the link between protein and muscle mass in males. Evidence suggests that there are gender-specific foods and male foods tend to be higher in protein (Bradbury and Nicolaou, 2012).

The maximum proportion of total energy that should come from alcohol is 5% (Dept_of_Health, 1991) however, the proportion of energy intake from alcohol in both genders in our sample was substantially lower, and also lower than the NDNS. Data was highly variable, with the majority of participants reporting that they did not drink alcohol; possibly for financial, ethnicity and religious reasons (74% of the cohort were BAME students) (Davoren et al., 2016; El Ansari et al., 2013). In this respect our sample did not represent the stereotypical student cohort (Breitenbach et al., 2016; Whatnall et al., 2019). However, some students may not have admitted to alcohol consumption, under-estimated intake or may have avoided alcohol altogether during the study.

Rich sources of vitamin A in the diet include retinol (liver and liver products, oily fish, eggs, milk and dairy products) and carotenes (fruit and vegetables) (PHE, 2018b). Further evidence that students in this study may not be consuming enough fruit and vegetables was the low intake of vitamin A compared with RNI and NDNS data. 76% of females and 83% of males achieved the RNI, whereas NDNS data showed RNI is readily met. Milk and milk products contribute to vitamin A intake. Calcium intakes meet 86% of RNI in females and 112% of RNI in males, which could indicate in females that milk and milk products are not major contributors of vitamin A. Other sources include oily fish and liver, which are not usually popular choices (PHE, 2018b).

Thiamin intakes were readily met. Main sources of thiamin include cereals and products made with white flour including bread, pasta, cakes, pasties and fortified breakfast cereals; all foods commonly consumed by students (Lupi et al., 2015). All flours in the UK must contain a minimum of 0.24 mg/100 g of thiamin (Gov.UK, 1998).

Male students consumed 154% of RNI values for iron, but on average, females only consumed 57% of iron RNI. For females, iron intakes (8.4 mg/d) were found to be lower than those of the NDNS (9.3 mg/d). Conversely, intakes in males were higher (13.4 mg/d) than the NDNS (11.6 mg/d). 54.8% of females were below the LRNI compared to 27% in the NDNS data. Total red and processed meat consumption in the UK is reported to be 77 g per day for males and 47 g per day for females (PHE, 2018b).

The logistic regression analyses revealed that intake of many micronutrients were significant predictors of excessive consumption, although in the majority of cases, the magnitude of association was low (Table 3). However, large associations in particular were recorded between fibre consumption and the prediction of excessive amounts of vitamins A and C: a unit change in protein consumption was associated with raised odds of 11% and 19% of exceeding DRVs for vitamin A and C respectively. A unit change in saturated fat consumption was also substantively associated with a 12% increase in odds of exceeding DRVs for sodium.

In conclusion, energy intakes were lower than the DRV. The percentage total energy values for protein, fat, saturated fat and carbohydrate exceeded DRVs but the percentage energy from alcohol was below the maximum 5%. The DRVs were met for vitamin C, thiamin, and sodium/salt. Iron and calcium intakes were met in males but not in females. Intakes for fibre and vitamin A were below the DRVs. Student data was comparable to the NDNS, with the exception of alcohol, fibre, vitamin A, calcium and sodium/salt, which were all lower than the NDNS. To address the findings observed in this research, universities should consider promoting a healthy campus in terms of food provision, convenience, variety and affordability: for example, in canteens and vending machines. Food products offered should also be culturally acceptable to the student population. In addition, universities should support activities during induction weeks to encourage choosing healthier options, particularly when snacking. Some students may have limited cooking skills, so universities could offer ‘cook and eat’ sessions and provide links to healthy eating resources and recipes.

## Availability of data and materials

Data is available from the authors on reasonable request.

## Authors' contributions

The study was designed by Helen Martin and Deborah Pufal, who collected the data. John Stephenson conducted the analysis. All authors contributed to the manuscript and revised and approved the final draft.

## Transparency declaration

The lead author affirms that this manuscript is an honest, accurate, and transparent account of the study being reported. The reporting of this work is compliant with STROBE guidelines. The lead author affirms that no important aspects of the study have been omitted and that any discrepancies from the study as planned have been explained.
